# Fetal exposure to bisphenol A as a risk factor for the development of childhood asthma: an animal model study

**DOI:** 10.1186/1476-069X-11-8

**Published:** 2012-02-21

**Authors:** Yoichi Nakajima, Randall M Goldblum, Terumi Midoro-Horiuti

**Affiliations:** 1 Departments of Pediatrics and Biochemistry and Molecular Biology, The University of Texas Medical Branch, 301 University Boulevard, Galveston, TX, 77555-0366, USA

**Keywords:** Animal model, Asthma, Bisphenol A, BPA, Environmental estrogen, Enzyme, Fetal exposure, Metabolism, Ovalbumin, Ugt2b1

## Abstract

**Background:**

The prevalence of asthma in industrialized countries has been increasing dramatically and asthma is now the most common chronic disease of children in the United States. The rapidity of the increase strongly suggests that changes in environmental exposures are the likely cause of this epidemic. Further, the early onset of allergic manifestations suggests that these exposures may act on the prenatal development of the immune system. We have focused on the potential effects of bisphenol A (BPA), a chemical pollutant with one of the largest productions, on the development of childhood asthma. We have reported that perinatal BPA exposure promotes the development of allergic asthma in a mouse model. The current study was designed to identify a critical period of BPA exposure and to begin elucidating the mechanisms for this susceptibility.

**Methods:**

Female BALB/c mice received 10 micro g/ml BPA in their drinking water from one week before pregnancy until the end of the study. Some of the pups were transferred in the first 48 h of life from their BPA-loaded mother to an unexposed mother, or vice versa. Half of the pups were sensitized with a low dose of the experimental allergen ovalbumin (OVA), the rest received PBS as an unsensitized controls. On day 22, the pups were challenged by inhalations of ovalbumin or PBS followed by quantification of eosinophils in and hyperreactivity of their airways, major indicators of experimental asthma in this classical mouse model. Hepatic expression of two isoforms of UDP-glucuronosyltransferase (Ugt) was quantified by quantitative RT-PCR at various ages.

**Results:**

Pups exposed to BPA in utero and through breast milk, or in utero only, displayed an asthma phenotype in response to their "suboptimal" allergic sensitization, whereas, pups only exposed to BPA postnatally from breast milk, did not. The expression of Ugt2b1, an isoform related to BPA clearance in rats, was not detectable in mouse fetuses and newborn pups, but increased by day 5 and approached adult levels by day 25.

**Conclusions:**

Prenatal exposures that produce environmentally relevant burdens of BPA, followed by postnatal allergic sensitization and challenges, promote the development of experimental allergic asthma. Delayed expression of BPA-metabolizing enzymes may explain, at least in part, the enhanced fetal susceptibility to this common environmental contaminant.

## Background

The production of the industrial chemical bisphenol A (BPA) is one of the largest in the world (approximately 2.4 billion pounds in 2007 [1]). BPA has been used for over 40 years to make hard plastic products, such as, baby bottles, food-storage containers, lining for food and beverage containers, and dental sealants. The estrogenic effects of BPA in experimental animals make it a potential endocrine disruptor [2,3]. In January 2010, the US Food and Drug Administration (FDA) changed its stance on BPA toxicity from "posed no risk for humans" to indicate that exposure to the chemical is of "some concern" for infants and children [4].

Since newborn humans and mice generally have a Th2 skewed pattern of immune responses, they are more prone to develop allergic diseases until "immune-maturing" infections promote a shift toward Th1 responses [5]. Like estradiol, environmental estrogen exposure in utero might influence the development of the immune system during important transition periods [6]. Endogenous and environmental estrogens promote Th2 responses in mice, as indicated by increased production of IL-4, transcription factor GATA-3, and MHC class II expression and decreasing IFN-γ production by CD4^+^CD8^+ ^thymocytes, naïve CD4^+ ^T cells, and spleen dendritic cells [7,8]. Early exposure to BPA is particularly associated with heighted inflammation. Prenatal [9] and early postnatal [10] exposure of mice promotes Th2 polarization (e.g. increased IL-4 and reduced IFN-γ). Prenatal exposure to BPA is also associated with reduced percentages of T regulatory CD4 + CD25+ cells [9] which are associated with atopy. We postulated that perinatal exposure to environmental estrogens might enhance or maintain the Th2 skewed pattern during this period of rapid immunologic transition.

We recently reported that a combination of pre-and postnatal exposures to maternally-derived BPA which produced environmentally relevant BPA burdens in mouse pups, promotes the development of allergic asthma in a mouse model [11]. Pups born to and nursed by mothers exposed to BPA in their drinking water responded to a "suboptimal" allergenic sensitization protocol by producing higher concentrations of serum IgE anti-ovalbumin (OVA) antibodies, numbers of bronchial eosinophils, and airway hyperreactivity (AHR), relative to pups born to and nursed by mothers that were not exposed to BPA.

We felt it was important to further define the critical period(s) during which BPA exposure might promote the development of childhood asthma and thus hypothesized that prenatal exposures to BPA were critical to promote the development of asthma. The dose of BPA we used produces internal doses of BPA in the mother mice and their pups that are similar to those in inadvertently-exposed humans [12]. To separate the prenatal and early-postnatal effect of BPA in our experimental model, some of the infant mice were transferred from BPA-loaded birth mothers to unexposed foster mothers, or vice versa, within 48 h after birth. After allergic sensitization and challenge, the pups were assessed for bronchial hyperreactivity. Further, we explore here metabolic mechanisms that might enhance the susceptibility of the mouse pups to BPA, by assessing the expression of a major enzyme that metabolizes BPA. Thus, we have tested the hypothesis that delayed expression of BPA metabolizing enzymes in the fetus enhances its exposure to transplacentally acquired, unconjugated (estrogenic) BPA.

## Methods

### Animals

Since human fetuses and young children are Th2 dominant, we chose for our studies BALB/c mice, which have been shown to have Th2 dominant immune responses [13]. Male and female BALB/c mice were obtained from Harlan (Houston, TX) and were housed in pathogen-free conditions in the animal research facility of the University of Texas Medical Branch (UTMB; Galveston, TX) in accordance with the National Institutes of Health and UTMB institutional guidelines for animal care. Each mother and her litter were housed separately and fed a casein-based diet (Research Diet, New Brunswick, NJ) to eliminate estrogenic exposure from the typical soy-based mouse diet from one week before BPA loading until the end of the study [11]. We used animal cages made of polysulfone (Tecniplast, Buguggiate, Italy) and bottled water, previously shown to be free of detectable levels of BPA [11].

### Experimental model of asthma

**Figure 1:**
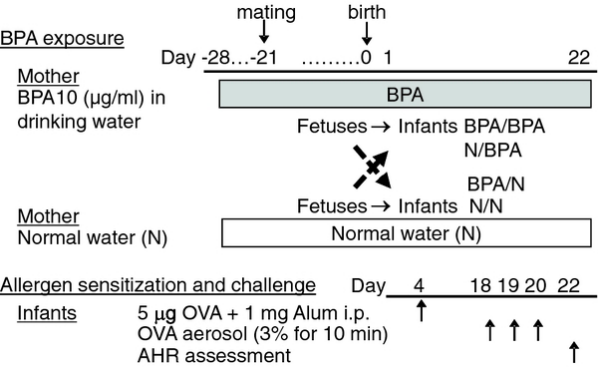
**Maternal BPA loading and OVA sensitization and challenge, and (B) AHR of the pups**. Some of the pups were transferred from BPA loaded to non-exposed mothers, and vice versa, prior to sensitization with OVA

Our protocol for BPA-loading and allergic sensitization, shown in Figure 1 was a modification of our previously published protocol [11]. Adult female mice were given 10 μg/ml of BPA (Sigma, St. Louis, MO) in their drinking water from one week before mating, until the end of the experiments. Some of the pups were transferred within 48 h after birth from BPA-loaded birth mother to unexposed foster mothers, and vice versa [14]. This manipulation resulted in four groups of pups, based on their BPA exposure, BPA-BPA: both prenatal and postnatal exposure to BPA, BPA-N: only prenatal exposure, N-BPA: only postnatal and N-N: no BPA exposure. Pups sensitized to OVA using our "suboptimal" protocol [11] formed the experimental groups and non-sensitized pups with the same BPA exposures served as controls for independent effects of BPA on lung function. Forty-eight h after challenging the pups with nebulized OVA, AHR to methacholine was assessed in all pups by the forced oscillation technique (flexiVent; SCIREQ, Montreal, Quebec, Canada) as we described [11].

### Quantifying the inflammatory cells in bronchoalveolar lavage (BAL) fluid

Immediately after sacrifice, cells in the lungs were recovered by flushing the isolated trachea with 0.5 mL PBS. Total leukocytes were counted using a hemocytometer. Eosinophil counts were calculated from differential cell counts on 150 μL of the fluid deposited onto glass slides, using a Cytospin 3 centrifuge (400 × g for 4 min; Shandon Lipshaw, Pittsburgh, PA) and stained with hematoxylin and eosin [11]. The results were expressed as the absolute number of the total cells and eosinophils recovered.

### Serum BPA

We quantified total BPA concentrations by ELISA (IBL America, Minneapolis, MN) in the sera from 22 day-old pups exposed to BPA both pre- and postnatally.

### Quantification of hepatic mRNA expression of Ugt2b1 and Ugt1a1

Gestational day (GD) 1 was considered the day when the vaginal plug was detected. Liver samples were harvested from untreated fetal mice on gestational day (GD) 17 and 19, infant pups on postnatal day (PND) 0, 5, 15 and 25 and adult mice after the age of 9 weeks. Liver samples were immediately stabilized by RNA later reagent (QIAGEN, Valencia, CA) and frozen at -80°C.

### Total RNA Isolation

Approximately 30 mg samples of liver were disrupted and homogenized in TRIZOL (Invitrogen, Carlsbad, CA) using 21 gauge needles and syringes. Total RNA was purified using RNeasy Mini Kit (QIAGEN, Valencia, CA). RNA was quantified by UV photometry at 260 nm and the purity and integrity were confirmed by their OD 260/280 ratio and visualization of 18S and 28S rRNA bands on the ethidium bromide gels.

### RT-Real-time PCR

We quantified mRNA expression for two isotypes of glucuronyl transferases in these livers using real-time PCR as we described [15]. cDNA was synthesized from total RNA using High-Capacity cDNA Reverse Transcription Kits (Applied Biosystems, Foster City, CA) according to the manufacturer's protocol. FAM dye-labeled TaqMan Gene Expression Assays, including gene-specific polymerase chain reaction (PCR) primers and dye-labeled probes, specific for mouse Ugt1a1 (Mm02603337_m1), Ugt2b1 (Mm00514184_m1), and mouse glyceraldehydes-3-phosphate dehydrogenase (Mm99999915_g1) were purchased from Applied Biosystems.

Real-time reactions composed of cDNA, gene-specific 20X TaqMan probes with Deoxynucleotide Solution Mix (New England BioLabs) and Taq DNA Polymerase (New England BioLabs) were performed in duplicate. Reaction conditions were as follows: initial setup at 50°C for 2 min and at 95°C for 10 min, followed by 45 cycles of 95°C for 15 sec, and 60°C for 1 min. Real-time reactions were performed on iQ5 Multicolor Real-time PCR Detection system (BIO-RAD) and data were obtained as Ct values. Data were analyzed by the ΔΔCt method using glyceraldehydes-3-phosphate dehydrogenase as an internal control for each sample. mRNA from a single adult male liver served as the reference.

### Statistical analysis

The pregnant mouse dam was the experimental unit for all experiments. Results for the pups from each dam are expressed as the mean ± SE. Statistical analysis was performed using one-way analysis of variance. Where differences between groups were present, the elements that differed were identified by the Student's *t*-test. Differences at *p *< 0.05 were defined as statistically significant.

## Results

### BPA exposure and bronchial hyperreactivity to methacholine

**Figure 2:**
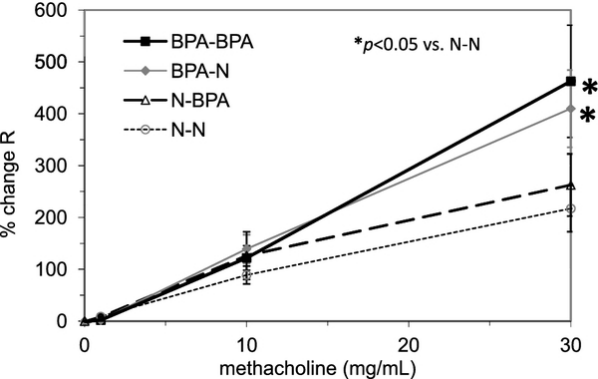
**AHR assessed with methacholine**. N: normal water. R: airway resistance. BPA-BPA: both prenatal and postnatal exposure to BPA, BPA-N: only prenatal exposure, N-BPA: only postnatal and N-N: no BPA exposure. Data are expressed as mean ± SE (n = 5-11 litters). There was no significant difference between any of the non OVA-sensitized groups. *p < 0.05 compared with BPA non exposed and OVA sensitized groups

Figure 2 shows that maternal exposure to BPA enhanced the development of AHR in the lungs of those pups that were exposed to BPA in utero only, and in utero and postnatally though breast milk, compared with pups that were only exposed to BPA postnatally, or not exposed to BPA at all (*p *< 0.05). Mice that were not sensitized with OVA did not show any significant effects of BPA exposure on their AHR (data not shown).

### Effects of BPA on airway inflammation

**Figure 3:**
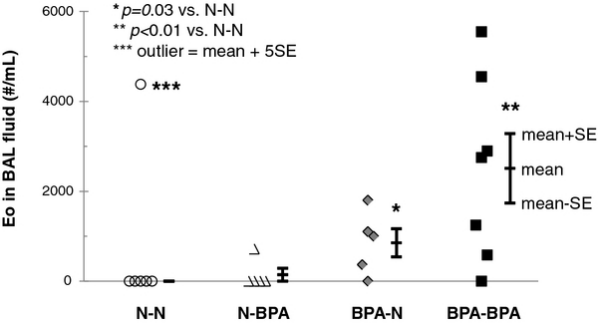
**BPA effects on OVA induced airway inflammation**. Eosinophil counts in BAL fluid 18 days after i.p. injection of OVA. Bars indicate the mean ± SE for groups. N = 4-9 litters. *p = 0.03 and **p < 0.01 compared with OVA-sensitized pups from non BPA exposed mothers. *** The eosinophil number in one BAL fluid was 5SE greater than the mean of the other samples. It was considered an outlier and not included in the statistics. There was no significant difference between any of the non OVA-sensitized groups

To determine whether BPA alters allergen-induced pulmonary inflammation, we quantified total cells and eosinophils in BAL fluid. We observed a significant increase in eosinophils in BAL fluids from OVA sensitized pups exposed to BPA only in utero, *p *= 0.03 vs. non BPA exposed group and those exposed in utero and postnatal BPA, *p *< 0.01 vs. the non BPA exposed group (Figure 3). There were no significant differences in total cell numbers between any groups.

### Expression of glucuronosyltransferases enzymes during pre-and postnatal development

**Figure 4:**
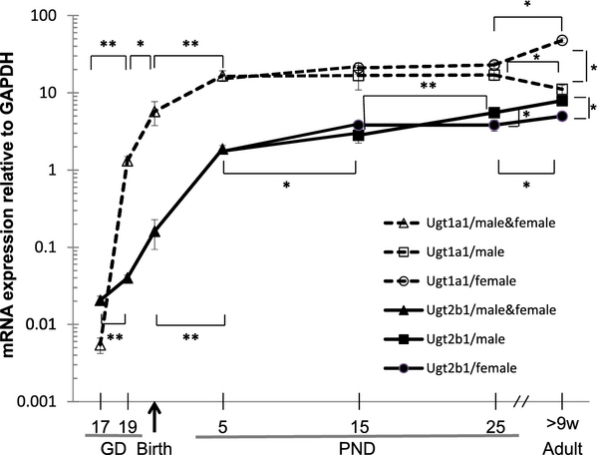
**Relative Ugt1a1 (A) and Ugt2b1 (B) expression as determined by real-time PCR on RNA purified from mouse livers**. Data are relative to standard mRNA from one adult male and computed by the ΔΔCt method. Data are mean ± SE (pup: n = 4-7 litters, adult; n = 10). *p < 0.05 and **p < 0.005 between groups.

As shown in Figure 4, the expression of mRNA for both isoforms of Utg was very low (around 0.1% of adult level) early in fetal development. However Ugt1a1, the main bilirubin metabolizing isoform, increased rapidly by gestational day 17 to achieve near adult levels at birth. In contrast, Ugt2b, an isoform related to BPA clearance in rats [16], increased much more slowly in utero and did not approach adult levels of expression until PND 5.

### Serum BPA in the pups

The concentrations of total BPA in the serum of 22 day-old pups that were born to exposed dams whose drinking water contained 10 μg/ml of BPA for a total of approximately 50 days (7 days before pregnancy, 21 days during pregnancy and 22 day postpartum) had a range of 29-39 (31 ± 4) ng/ml.

## Discussion

We originally embarked on studies of environmental estrogens in asthma pathogenesis [17,18] based on extensive epidemiological evidence that there are major differences in asthma susceptibility between men and women during different life stages, with males predominating (male: female ratio = 3:1) from infancy to age 14 and females predominating (1:3) from ages 18 to 34 [19]. Further, hormone replacement therapy (especially with estrogens) in women enhances both new onset [20] and worsening of previously quiescent asthma [21], suggesting that estrogenic agent may play a role in the gender difference in asthma prevalence. A molecular basis for BPA's effects on immune events in asthma was suggested by our and our colleagues' finding that immune cells, including mast cells and lymphocytes from humans and mice, expressed estrogen receptor (ER)α, but not ERβ [8,17]. Expression of ERα was required for environmental estrogens, including nonylphenol, an alkylphenol, to enhance basophil degranulation [18]. BPA also has a high affinity to estrogen-related receptor (ERR)γ [22], but ERRγ has yet to be described on any immune cells. We also detected G-protein coupled receptor 30 (GPR30) on mast cells (unpublished observation from Dr. Peter Thomas). Androgen receptor (AR) was detected by others on mast cells and lymphocytes [23,24]. Aryl hydrocarbon receptor (AhR) was also detected on lymphocytes, but not on mast cells, but the relationship between AHR, ERs and estrogens is quite complex.

In our previous study, we demonstrated that pups that were born to and nursed by dams that were exposed to BPA had an enhanced propensity to develop asthma after an intentionally "suboptimal" allergic sensitization during infancy [11,25]. In the current study, we transferred some pups from BPA-loaded birth mothers to unexposed foster mothers and vice versa, within 48 h after their birth [14]. These transfers created four groups of pups that allowed us to test our hypothesis that the prenatal exposures to BPA were required to promote the development of allergic asthma in our mouse model. Consistent with this hypothesis, only the pups exposed to BPA prenatally demonstrated enhanced airway inflammation and hyperreactivity. These results strongly suggest that prenatal life is a critical period, during which BPA exposure promotes the development of asthma. However, as we previously observed [11], mice that are exposed to BPA, but not sensitized to OVA did not develop an asthma phenotype, suggesting that prenatal BPA exposure enhances the allergic component of asthma pathogenesis.

We chose the concentration of BPA to feed dams based on a previous report that this dose, fed for a similarly short period of time, produces a BPA burden (internal dose) with a mean of 10-25 ng/g tissue in brain, kidney, liver and testis, as measured by GC-MS analysis [12]. These burdens are similar to those in human samples, which contained BPA at concentrations of up to 104.9 ng/g tissue [26]. To confirm the relevance of this dose in our mouse model to environmental BPA exposures of humans, we quantified total BPA concentrations in the sera of pups that were exposed both pre-and postnatally to maternal BPA. The range of BPA concentrations in the sera of the mouse pups 29-39 ng/ml was only slightly greater than the upper range found in two studies of plasma BPA in pregnant women (0.5-22.3 and 0.3-18.9 ng/ml) from the US and Germany [26,27]. The large variability of BPA concentrations in human cord blood, likely due to different levels of exposures of their mothers, suggests that some infants may have in utero BPA burdens similar or greater than those of our experimentally-exposed mice.

Next, we tested the hypothesis that delayed development of BPA metabolizing enzymes in the fetus enhances fetal exposure to transplacental, unconjugated (estrogenic) BPA and its immunotoxicity. Steroid hormones, including environmental estrogens like BPA, are largely metabolized by glucuronidation by UGT and then excreted through the urine [28]. The mammalian UGT gene superfamily currently has 117 members that can be divided into four families, UGT1, UGT2, UGT3 and UGT8 [29]. The UGT1A group share homology between species, but the UGT2B group is only homologous between rats UGT2B1 and mice Ugt2b1. In human liver, UGT2B15 showed the highest activity for BPA glucuronidation at low (1.0 μM ≈ 0.2 ng/ml) and high (20 μM ≈ 5 ng/ml) substrate concentration, while UGT1A1, UGT1A3, UGT1A9, UGT2B4 and UGT2B7 were also capable of catalyzing BPA glucuronidation, though only at higher concentration of BPA (20 μM) [30]. In rat liver, BPA is mainly glucuronidated by UGT2B1 [31]. Because rat UGT2B1 shares homology with mouse Ugt2b1, it is likely that BPA is mainly glucuronidated by Ugt2b1 in mouse liver. Thus, we assessed the expression of Ugt2b1 in mouse fetuses as a possible mechanism of BPA's enhancement of asthma development in mouse pups. Another isoform Ugt1a1, a major enzyme for glucuronidation of bilirubin in newborn rats, was used as a control for hepatic expression of Ugts. Our results indicated that the hepatic expression of Ugt2b1, the isoform related to BPA clearance in rats [16] was very low throughout the fetal period in our mice, but increased rapidly after birth. It is possible that this postnatal increase in Ugt2b1 expression may have protected the pups from postnatal effects of BPA-exposure. The expression of Ugt1a1, the main bilirubin-conjugating isoforms developed more rapidly in utero and reached near adult levels at birth, indicating that the expression of some isoforms are easily detected in the fetal livers of the mice we studied. Together these results are consistent with the hypothesis that unconjugated BPA may accumulate during the prenatal period and act as an endocrine disruptor that alters the course of the rapid development of the immune system.

The relevance of these metabolic findings in our mouse model to BPA exposure of the human fetus and infants has yet to be tested directly. However, expression of Ugt2B15 [30], which is responsible for glucuronidation of BPA in human liver was not detected at 20 weeks of gestation [32] and glucuronidation of another estrogen, estrone, by fetal and neonatal human liver microsomes was approximately 30% of adult activity [33]. Furthermore, high level expression of β-glucuronidase enzymes in the human fetus and placenta, which can deconjugate BPA glucuronide [34], could also enhance free BPA concentrations in mothers and their fetuses. Sulfotransferase (SULT)1A1, a human enzyme that also metabolizes BPA [35] was detected in human liver from early gestation [36]. However, high level expression of sulfatases and β-glucuronidase enzymes, which deconjugate BPA glucuronide and sulfate are also present in the human fetus and placenta [34,37], which could increase the concentration of free BPA in mothers and their fetuses. Understanding the dynamics of BPA in the placenta, fetus and neonate will require careful quantification of BPA and its conjugates in relevant tissues and fluids.

While the low level of prenatal expression of Ugt2b1 we describe here could allow accumulation of unconjugated BPA in the pups, and thereby, enhance BPA's effects on the development of asthma, other differences between pre-and postnatal exposures could also be involved. For instance, the route of BPA exposure (systemic for fetus vs. intestinal for infants), maturation state of the immune system and susceptibility to epigenetic alteration, alone or induced by high level exposure to maternal hormones might also enhance the impact to prenatal BPA exposures on the development of allergic asthma. Similarly, the concentration of BPA in the dam's milk may have limited the BPA exposure in the pups which did not receive maternal BPA prenatally.

## Conclusions

In conclusion, the results of the experiments we describe here provide the first evidence that the prenatal period may be a critical one for BPA to promote the development of asthma. Developmental delays in the expression of the BPA-metabolizing enzymes may be at least one reason for this susceptibility in our mouse model. Studies of environmental exposures to BPA of the human fetus may help to explain the ongoing epidemic of childhood asthma.

## Abbreviations

AHR: Airway hyperresponsiveness; BPA: Bisphenol A; FDA: Food and Drug Administration; GD: Gestational day; Ig: Immunoglobulin; OVA: Ovalbumin; PND: Post natal day; RT-PCR: Reverse transcription-polymerase chain reaction; SE: Standard error; SULT: Sulfatase; Th: T helper; Ugt: Uridine 5'-diphosphate glucuronosyltransferases

## Competing interests

The authors declare that they have no competing interests.

## Authors' contributions

TMH carried out the mouse model study. YN carried out the BPA measurement and molecular gene analysis. All authors participated in the design and analyses of the studies, wrote, read and approved the final manuscript.

## References

[B1] U.S.Environmental Protection Agency Bisphenol A Action Plan (CASRN 80-05-7) 2010 http://www.epa.gov/oppt/existingchemicals/pubs/actionplans/bpa_action_plan.pdf

[B2] Alyea RA Watson CS Differential regulation of dopamine transporter function and location by low concentrations of environmental estrogens and 17beta-estradiol Environ Health Perspect 2009 117 778 783 10.1289/ehp.0800026 PMC2685841 19479021

[B3] Myers JP Vom Saal FS Akingbemi BT Arizono K Belcher S Colborn T Why public health agencies cannot depend on good laboratory practices as a criterion for selecting data: the case of bisphenol A Environ Health Perspect 2009 117 309 315 10.1289/ehp.0800173 PMC2661896 19337501

[B4] FDA update Update on Bisphenol A for Use in Food Contact Applications 2010 http://www.fda.gov/NewsEvents/PublicHealthFocus/ucm197739.htm

[B5] Martino DJ Prescott SL Silent mysteries: epigenetic paradigms could hold the key to conquering the epidemic of allergy and immune disease Allergy 2010 65 7 15 10.1111/j.1398-9995.2009.02186.x 19796189

[B6] Chalubinski M Kowalski ML Endocrine disrupters-potential modulators of the immune system and allergic response Allergy 2006 61 1326 1335 10.1111/j.1398-9995.2006.01135.x 17002710

[B7] Iwata M Eshima Y Kagechika H Miyaura H The endocrine disruptors nonylphenol and octylphenol exert direct effects on T cells to suppress Th1 development and enhance Th2 development Immunol Lett 2004 94 135 139 10.1016/j.imlet.2004.04.013 15234545

[B8] Lambert KC Curran EM Judy BM Milligan GN Lubahn DB Estes DM Estrogen Receptor alpha (ER{alpha}) Deficiency in Macrophages Results in Increased Stimulation of CD4+ T Cells while 17{beta}-Estradiol Acts through ER{alpha} to Increase IL-4 and GATA-3 Expression in CD4+ T Cells Independent of Antigen Presentation J Immunol 2005 175 5716 5723 10.4049/jimmunol.175.9.5716 16237062

[B9] Yan H Takamoto M Sugane K Exposure to Bisphenol A prenatally or in adulthood promotes T(H)2 cytokine production associated with reduction of CD4CD25 regulatory T cells Environ Health Perspect 2008 116 514 519 10.1289/ehp.10829 PMC2290985 18414636

[B10] Sawai C Anderson K Walser-Kuntz D Effect of bisphenol A on murine immune function: modulation of interferon-gamma, IgG2a, and disease symptoms in NZB × NZW F1 mice Environ Health Perspect 2003 111 1883 1887 10.1289/ehp.6359 PMC1241761 14644661

[B11] Midoro-Horiuti T Tiwari R Watson CS Goldblum RM Maternal exposure to bisphenol A enhances susceptibility to experimental allergic asthma in their pups Env Health Perspectives 2010 118 273 277 10.1289/ehp.0901259 PMC2831929 20123615

[B12] Kabuto H Amakawa M Shishibori T Exposure to bisphenol A during embryonic/fetal life and infancy increases oxidative injury and causes underdevelopment of the brain and testis in mice Life Sci 2004 74 2931 2940 10.1016/j.lfs.2003.07.060 15051418

[B13] Becker M Reuter S Friedrich P Doener F Michel A Bopp T Genetic variation determines mast cell functions in experimental asthma J Immunol 2011 186 7225 7231 10.4049/jimmunol.1100676 21572035

[B14] Leme AS Hubeau C Xiang Y Goldman A Hamada K Suzaki Y Role of breast milk in a mouse model of maternal transmission of asthma susceptibility J Immunol 2006 176 762 769 10.4049/jimmunol.176.2.762 16393959

[B15] Nakajima Y Tsuge I Kondo Y Komatsubara R Hirata N Kakami M Up-regulated cytokine-inducible SH2-containing protein expression in allergen-stimulated T cells from hen's egg-allergic patients Clin Exp Allergy 2008 38 1499 1506 10.1111/j.1365-2222.2008.03030.x 18647318

[B16] Matsumoto J Yokota H Yuasa A Developmental increases in rat hepatic microsomal UDP-glucuronosyltransferase activities toward xenoestrogens and decreases during pregnancy Environ Health Perspect 2002 110 193 196 10.1289/ehp.02110193 PMC1240735 11836149

[B17] Zaitsu M Narita S Lambert KC Grady JJ Estes DM Curran EM Estradiol activates mast cells via a non-genomic estrogen receptor-alpha and calcium influx Mol Immunol 2007 44 1987 1995 10.1016/j.molimm.2006.09.030 PMC2603032 17084457

[B18] Narita S Goldblum RM Watson CS Brooks EG Estes DM Curran EM Environmental estrogens induce mast cell degranulation and enhance IgE-mediated release of allergic mediators Env Health Perspectives 2007 115 48 52 10.1289/ehp.9378 PMC1797832 17366818

[B19] Vollmer WM Osborne ML Buist AS 20-year trends in the prevalence of asthma and chronic airflow obstruction in an HMO Am J Respir Crit Care Med 1998 157 1079 1084 10.1164/ajrccm.157.4.9704140 9563722

[B20] Barr RG Wentowski CC Grodstein F Somers SC Stampfer MJ Schwartz J Prospective study of postmenopausal hormone use and newly diagnosed asthma and chronic obstructive pulmonary disease Arch Intern Med 2004 164 379 386 10.1001/archinte.164.4.379 14980988

[B21] Lieberman D Kopernik G Porath A Lazer S Heimer D Sub-clinical worsening of bronchial asthma during estrogen replacement therapy in asthmatic post-menopausal women Maturitas 1995 21 153 157 10.1016/0378-5122(94)00890-j 7752953

[B22] Okada H Tokunaga T Liu X Takayanagi S Matsushima A Shimohigashi Y Direct evidence revealing structural elements essential for the high binding ability of bisphenol A to human estrogen-related receptor-gamma Environ Health Perspect 2008 116 32 38 10.1289/ehp.10587 PMC2199305 18197296

[B23] Chen W Beck I Schober W Brockow K Effner R Buters JT Human mast cells express androgen receptors but treatment with testosterone exerts no influence on IgE-independent mast cell degranulation elicited by neuromuscular blocking agents Exp Dermatol 2010 19 302 304 10.1111/j.1600-0625.2009.00969.x 19758318

[B24] Olson BM McNeel DG CD8+ T cells specific for the androgen receptor are common in patients with prostate cancer and are able to lyse prostate tumor cells Cancer Immunol Immunother 2011 60 781 792 10.1007/s00262-011-0987-5 PMC3319721 21350948

[B25] Hamada K Suzaki Y Goldman A Ning YY Goldsmith C Palecanda A Allergen-independent maternal transmission of asthma susceptibility J Immunol 2003 170 1683 1689 10.4049/jimmunol.170.4.1683 12574331

[B26] Schonfelder G Wittfoht W Hopp H Talsness CE Paul M Chahoud I Parent bisphenol A accumulation in the human maternal-fetal-placental unit Environ Health Perspect 2002 110 A703 A707 10.1289/ehp.110-1241091 PMC1241091 12417499

[B27] Padmanabhan V Siefert K Ransom S Johnson T Pinkerton J Anderson L Maternal bisphenol-A levels at delivery: a looming problem? J Perinatol 2008 28 258 263 10.1038/sj.jp.7211913 PMC4033524 18273031

[B28] Mackenzie PI Rodbourne L Stranks S Steroid UDP Glucuronosyltransferases J Steroid Biochem Molec Biol 1992 43 1099 1105 10.1016/0960-0760(92)90338-J 22217855

[B29] Mackenzie PI Bock KW Burchell B Guillemette C Ikushiro S Iyanagi T Nomenclature update for the mammalian UDP glycosyltransferase (UGT) gene superfamily Pharmacogenet Genomics 2005 15 677 685 10.1097/01.fpc.0000173483.13689.56 16141793

[B30] Hanioka N Naito T Narimatsu S Human UDP-glucuronosyltransferase isoforms involved in bisphenol A glucuronidation Chemosphere 2008 74 33 36 10.1016/j.chemosphere.2008.09.053 18990428

[B31] Yokota H Iwano H Endo M Kobayashi T Inoue H Ikushiro S Glucuronidation of the environmental oestrogen bisphenol A by an isoform of UDP-glucuronosyltransferase, UGT2B1, in the rat liver Biochem J 1999 340 Pt 2 405 409 PMC1220264 10333482

[B32] Strassburg CP Strassburg A Kneip S Barut A Tukey RH Rodeck B Developmental aspects of human hepatic drug glucuronidation in young children and adults Gut 2002 50 259 265 10.1136/gut.50.2.259 PMC1773113 11788570

[B33] de Wildt SN Kearns GL Leeder JS van den Anker JN Glucuronidation in humans. Pharmacogenetic and developmental aspects Clin Pharmacokinet 1999 36 439 452 10.2165/00003088-199936060-00005 10427468

[B34] Miki Y Nakata T Suzuki T Darnel AD Moriya T Kaneko C Systemic distribution of steroid sulfatase and estrogen sulfotransferase in human adult and fetal tissues J Clin Endocrinol Metab 2002 87 5760 5768 10.1210/jc.2002-020670 12466383

[B35] Nishiyama T Ogura K Nakano H Kaku T Takahashi E Ohkubo Y Sulfation of environmental estrogens by cytosolic human sulfotransferases Drug Metab Pharmacokinet 2002 17 221 228 10.2133/dmpk.17.221 15618673

[B36] Duanmu Z Weckle A Koukouritaki SB Hines RN Falany JL Falany CN Developmental expression of aryl, estrogen, and hydroxysteroid sulfotransferases in pre- and postnatal human liver J Pharmacol Exp Ther 2006 316 1310 1317 10.1124/jpet.105.093633 16339912

[B37] Lucier GW Sonawane BR McDaniel OS Glucuronidation and deglucuronidation reactions in hepatic and extrahepatic tissues during perinatal development Drug Metab Dispos 1977 5 279 287 17527

